# Peripheral blood and bone marrow responses under stress of cypermethrin in albino rats

**DOI:** 10.2478/intox-2014-0006

**Published:** 2014-07-16

**Authors:** Sunita Pande, Prabhu Narain Saxena, Brijender Bhushan, Nishi Saxena

**Affiliations:** 1 Maharani Shri Jaya Government Post Graduate College, Bharatpur, India; 2 Toxicology Laboratory, Department of Zoology, School of Life Sciences, Dr. B.R. Ambedkar University, Khandari Campus, Agra, India

**Keywords:** pyrethroid, blood, toxicity, contamination, environment

## Abstract

Pyrethroids, commercially available pesticides, are greatly in use these days, and thus they carry considerable chances of contaminating various ecosystems. Haematotoxicity of cypermethrin, a broadly used type II pyrethroid, has been assessed in the present study. Selected parameters included determination of total RBC count, haemoglobin concentration (Hb conc.), packed cell volume (PCV), mean corpuscular volume (MCV), mean corpuscular haemoglobin (MCH), mean corpuscular haemoglobin concentration (MCHC), erythrocyte sedimentation rate (ESR), total leukocyte count (TLC), differential leukocyte count (DLC), along with qualitative analysis of blood and bone marrow. Of these parameters, those showing significant decline following cypermethrin intoxication included total RBC count, Hb conc., PCV, MCV, MCH, whereas non-significant decrease was observed in the case of MCHC. ESR, TLC and DLC, on the other hand, increased significantly following cypermethrin intoxication. Qualitative changes included altered red cell morphology such as microcystosis, appearance of stomatocytes, poikilocytosis, giant platelet formation, etc. in peripheral blood and increased erythroid precursors in bone marrow of treated rats. These parameters were however normalised following twenty-two days of recovery phase.

## Introduction

The use of synthetic pesticides has been increasing considerably pertaining to enhanced global food demands, vector-borne diseases, pests and their genetically modified resistant species (Sayim *et al.*, 2005; Bhushan *et al.*, 2013a). Pyrethroids represent a class of broad spectrum insecticides, finding their rich utility among all categories of users. Synthetic pyrethroids, the synthetic analogues artificially designed by modifying the basic pyrethrin structure, presently account for about 30% of the insecticidal market worldwide (Soderlund *et al.*, 2002; Spencer and Sham, 2005; Addy-Orduna *et al.*, 2011; Bhushan *et al.,* 2013b). These pesticides are regarded to be comparatively safe, easily biodegradable and target specific, yet their excessive use can cause serious toxicological manifestations in non-target organisms by very many routes through interference and accumulation in various food chains (Rana *et al.*, 2008; Assayed *et al.*, 2010, Corcellas *et al.,* 2012; Bhushan *et al.*, 2013c). Synthetic pyrethroids fall under two categories, viz. type I and type II, based on the absence and presence of the alpha-cyano group, as well as on neurophysiological alterations in animals following intoxication (Singh *et al.,* 2009; Bhushan *et al.*, 2010). Cypermethrin is a broadly used type II pyrethroid pesticide having a broad spectrum of application, which in turn enhances its non-target toxicity (Aldana *et al.*, 2001; Bhushan *et al* 2013b).

The present study has therefore been aimed at the evaluation of haematotoxicity of cypermethrin through analysis of total RBC count, haemoglobin concentration, PCV, MCV, MCH, MCHC, ESR, TLC, DLC, along with qualitative analysis of peripheral blood and bone marrow.

## Material and Methods

### Experimental animal rearing and maintenance

The present study was conducted on seventy-two male albino rats, *Rattus norvegicus*, weighing 110±20 g, eight weeks in age, selected from an inbred colony per the ethical committee of the Department of Zoology, Dr. B.R. Ambedkar University, Agra. The experimental animals were provided standard rat pellet food and water *ad libitum*. The rats were acclimatised to laboratory conditions for two weeks prior to experimentation. Then they were divided into six treatment sets, containing six rats each, which were orally administered cypermethrin mixed with groundnut oil by gavage tube. Controls were also run simultaneously and fed groundnut oil.

### Experimental compound

Cypermethrin (≈95% purity) obtained from Rallis India Ltd., Mumbai was used as experimental compound in the present study and its LD_50_ was calculated (Finney, 1971) as 433.6 mg/kg b.wt. (Pande, 2001; Bhushan *et al.*, 2013b).

### Dose administration and sample collection

Technical grade of cypermethrin was orally administered to experimental albino rats of respective sub-sets as per acute [300 mg/kg b.wt./rat for one day] and sub-chronic (10.7/7, 10.7/14, 10.7/21 and 10.7/28 mg/kg b. wt. for 7, 14, 21 and 28 days) doses of cypermethrin. Animals of the sixth treatment set were administered a dose of 10.7 mg/kg b. wt./rat for 28 days and thereafter left untreated for the next 22 days. Controls corresponding to each treatment set were run simultaneously. These albino rats were etherised after predetermined time intervals to collect blood from the heart ventricle through a hypodermic needle to analyse various haematological parameters, viz. total red blood cell count (TRBC count), erythrocyte sedimentation rate (ESR), total leukocyte count (TLC), differential leukocyte count (DLC), haemoglobin concentration (Hb. Conc.), packed cell volume (PCV), mean corpuscular volume (MCV), mean corpuscular haemoglobin (MCH) and mean corpuscular haemoglobin concentration (MCHC) by Beckman Coulter analyzer.

### Qualitative changes

Bone marrow and blood smears were prepared and stained with Leishman stain for morphological evaluation.

### Statistical Analysis

Data obtained from haematological studies were statistically analysed for significant difference, if any, by Student's “t” test by the software SPSS 11.5 for windows.

## Results

### Haematological changes

Of the haematological parameters observed in the experimental groups and compared with respective control groups, total RBC count, haemoglobin concentration, packed cell volume, mean corpuscular volume, and mean corpuscular haemoglobin decreased significantly, whereas a non-significant decrease was observed in case of mean corpuscular haemoglobin concenteration. Erythrocyte sedimentation rate, total leukocyte count and differential leukocyte count showed an increasing trend following cypermethrin intoxication. These parameters became normalised after the recovery phase of twenty-two days (Table 1–10).

**Table 1:** Effect of Cypermethrin on Total RBC Count (million/mm^3^) in *Rattus norvegicus.*

S.No.	Treatment	Treatment duration (Days)	No. of rats treated	Dose (mg/kg b.w.)	Range	Mean±S.E.	% change	Significance level
1.	Control*	1	6	–	6.74–6.83	6.77±0.02	−1.5	*p<*0.05
2.	Acute	1	6	300	6.60–6.76	6.67±0.07		
3.	Control*	7	6	–	6.68–6.94	6.83±0.04	−1.02	*p<*0.05
4.	Subchronic	7	6	10.7	6.52–6.86	6.76±0.05		
5.	Control*	14	6	–	6.79–6.93	6.87±0.02	−4.22	*p<*0.01
6.	Subchronic	14	6	10.7	6.10–7.05	6.58±0.02		
7.	Control*	21	6	–	6.78–6.95	6.87±0.03	−4.37	*p<*0.001
8.	Subchronic	21	6	10.7	5.33–6.72	6.57±0.21		
9.	Control*	28	6	–	6.75–6.98	6.84±0.03	−4.68	*p<*0.001
10.	Subchronic	28	6	10.7	6.42–6.70	6.52±0.06		

Recovery group**								

a.	Control*	–	6	–	6.74–6.83	6.77±0.02	±1.46	*p*>0.05
b.	Treated	–	6	10.7	6.79–6.93	6.87±0.02		

* Controls were given groundnut oil only

** Recovery group were given oil (a) and cypermethrin (b) treatment for 28 days and effects assessed after 60 days

**Table 2:** Effect of Cypermethrin on Haemoglobin Concentration (g/dl) in *Rattus norvegicus.*

S.No.	Treatment	Treatment duration (Days)	No. of rats treated	Dose (mg/kg b.w.)	Range	Mean±S.E.	% change	Significance level
1.	Control*	1	6	–	12.2–13.1	12.6±0.7	−4.28	*p<*0.01
2.	Acute	1	6	300	10.8–13.4	12.06±0.12		
3.	Control*	7	6	–	11.0–13.8	12.4±0.16	−3.22	*p<*0.01
4.	Subchronic	7	6	10.7	11.8–12.3	12.0±0.08		
5.	Control*	14	6	–	10.0–14.0	12.5±0.22	−7.2	*p<*0.05
6.	Subchronic	14	6	10.7	11.0–12.5	11.6±0.24		
7.	Control*	21	6	–	10.8–12.6	12.2±0.15	−4.92	*p<*0.05
8.	Subchronic	21	6	10.7	9.2–11.4	11.6±0.43		
9.	Control*	28	6	–	10.8–12.8	12.3±0.17	−6.5	*p<*0.01
10.	Subchronic	28	6	10.7	10.2–12.1	11.5±0.31		

Recovery group**								

a.	Control*	–	6	–	11.0–13.0	12.5±0.22	±0.18	*p*>0.05
b.	Treated	–	6	10.7	11.2–13.1	12.2±0.7		

* Controls were given groundnut oil only

** Recovery group were given oil (a) and cypermethrin (b) treatment for 28 days and effects assessed after 60 days

**Table 3:** Effect of Cypermethrin on Packed Cell Volume (%) in Blood of *Rattus norvegicus.*

S.No.	Treatment	Treatment duration (Days)	No. of rats treated	Dose (mg/kg b.w.)	Range	Mean±S.E.	% change	Significance level
1.	Control*	1	6	–	38–44	41.83±0.82	−5.97	*p<*0.05
2.	Acute	1	6	300	36–43	39.33±0.23		
3.	Control*	7	6	–	42–44	42.67±0.36	−2.34	*p>*0.05
4.	Subchronic	7	6	10.7	38–46	41.67±0.36		
5.	Control*	14	6	–	40–46	42.5±0.73	−5.5	*p<*0.05
6.	Subchronic	14	6	10.7	38–42	40.16±0.71		
7.	Control*	21	6	–	38–45	43.33±0.54	−3.85	*p<*0.05
8.	Subchronic	21	6	10.7	36–44	41.66±0.78		
9.	Control*	28	6	–	38–45	42.67±0.54	−5.08	*p<*0.05
10.	Subchronic	28	6	10.7	38–42	40.5±0.68		

Recovery group**								

a.	Control*	–	6	–	37–44	42.67±0.36	−1.66	*p>*0.05
b.	Treated	–	6	10.7	36–45	41.96±0.54		

* Controls were given groundnut oil only

** Recovery group were given oil (a) and cypermethrin (b) treatment for 28 days and effects assessed after 60 days

**Table 4:** Effect of Cypermethrin on Mean Corpuscular Volume (µ^3^) in *Rattus norvegicus.*

S.No.	Treatment	Treatment duration (Days)	No. of rats treated	Dose (mg/kg b.w.)	Range	Mean±S.E.	% change	Significance level
1.	Control*	1	6	–	58.6–65.8	61.8±1.29	−2.42	*p>*0.05
2.	Acute	1	6	300	56.7–61.9	60.3±1.13		
3.	Control*	7	6	–	60.1–64.7	62.5±0.60	−1.44	*p>*0.05
4.	Subchronic	7	6	10.7	60.0–63.7	61.6±0.63		
5.	Control*	14	6	–	59.2–65.2	61.8±1.10	−0.16	*p>*0.05
6.	Subchronic	14	6	10.7	59.6–63.9	61.7±0.71		
7.	Control*	21	6	–	61.6–66.5	64.6±0.89	−6.34	*p<*0.01
8.	Subchronic	21	6	10.7	57.2–63.2	60.5±0.91		
9.	Control*	28	6	–	58.5–68.7	62.3±1.08	−3.53	*p<*0.01
10.	Subchronic	28	6	10.7	59.2–63.4	60.1±1.07		

Recovery group**								

a.	Control*	–	6	–	60.1–63.9	62.5±0.60	−1.12	*p>*0.05
b.	Treated	–	6	10.7	58.6–65.8	61.8±1.29		

* Controls were given groundnut oil only

** Recovery group were given oil (a) and cypermethrin (b) treatment for 28 days and effects assessed after 60 days

**Table 5:** Effect of Cypermethrin on Mean Corpuscular Haemoglobin (pg) in *Rattus norvegicus*.

S.No.	Treatment	Treatment duration (Days)	No. of rats treated	Dose (mg/kg b.w.)	Range	Mean±S.E.	% change	Significance level
1.	Control*	1	6	–	16.08–22.29	18.56±0.39	−1.96	*p>*0.05
2.	Acute	1	6	300	14.88–21.91	18.21±0.21		
3.	Control*	7	6	–	13.21–24.76	18.58±0.26	−1.43	P>0.05
4.	Subchronic	7	6	10.7	16.88–22.93	18.31±0.18		
5.	Control*	14	6	–	13.12–23.85	18.19±0.33	−2.69	P<0.05
6.	Subchronic	14	6	10.7	14.36–26.61	17.70±0.35		
7.	Control*	21	6	–	10.07–38.58	18.14±0.24	−2.92	P>0.05
8.	Subchronic	21	6	10.7	10.99–27.86	17.30±0.14		
9.	Control*	28	6	–	16.59–18.60	17.91±0.23	−7.03	P>0.01
10.	Subchronic	28	6	10.7	10.50–23.0	16.65±0.18		

Recovery group**								

a.	Control*	–	6	–	13.71–28.51	17.64±0.35	±2.04	P>0.05
b.	Treated	–	6	10.7	14.29–28.56	18.0±0.26		

* Controls were given groundnut oil only

** Recovery group were given oil (a) and cypermethrin (b) treatment for 28 days and effects assessed after 60 days

**Table 6:** Effect of Cypermethrin on Mean Corpuscular Haemoglobin Concentration (g/dl) in *Rattus norvegicus.*

S.No.	Treatment	Treatment duration (Days)	No. of rats treated	Dose (mg/kg b.w.)	Range	Mean±S.E.	% change	Significance level
1.	Control*	1	6	–	20.18–32.0	29.76±0.61	±3.06	P>0.05
2.	Acute	1	6	300	20.0–38.79	30.67±0.29		
3.	Control*	7	6	–	22.9–36.47	29.14±0.42	−1.44	P>0.05
4.	Subchronic	7	6	10.7	26.61–29.75	28.72±0.37		
5.	Control*	14	6	–	23.57–39.21	29.42±0.50	−1.57	P>0.05
6.	Subchronic	14	6	10.7	25.42–36.52	28.97±0.47		
7.	Control*	21	6	–	20.81–49.52	28.01±0.43	−3.07	P>0.05
8.	Subchronic	21	6	10.7	23.55–39.18	27.15±0.92		
9.	Control*	28	6	–	26.81–39.73	28.78±0.72	−1.49	P>0.05
10.	Subchronic	28	6	10.7	20.84–40.0	28.35±0.47		

Recovery group**								

a.	Control*	–	6	–	21.93–38.47	29.14±0.42	±0.96	P>0.05
b.	Treated	–	6	10.7	19.57–48.29	29.42±0.50		

* Controls were given groundnut oil only

** Recovery group were given oil (a) and cypermethrin (b) treatment for 28 days and effects assessed after 60 days

**Table 7:** Effect of Cypermethrin on Erythrocyte Sedimentation Rate (ESR) (mm 1^st^ hour) in *Rattus norvegicus.*

S.No.	Treatment	Treatment duration (Days)	No. of rats treated	Dose (mg/kg b.w.)	Range	Mean±S.E.	% change	Significance level
1.	Control*	1	6	–	1–2	1.33±0.23	±50.37	P>0.05
2.	Acute	1	6	300	1–3	2.0±0.39		
3.	Control*	7	6	–	2–3	2.33±0.23	−21.46	P>0.05
4.	Subchronic	7	6	10.7	1–3	1.83±0.34		
5.	Control*	14	6	–	2–2	2.0±0.0	−25.0	P>0.05
6.	Subchronic	14	6	10.7	1–2	1.5±0.24		
7.	Control*	21	6	–	1–2	1.33±0.26	±75.18	P<0.05
8.	Subchronic	21	6	10.7	2–3	2.33±0.23		
9.	Control*	28	6	–	2–2	2.0±0.0	0	P>0.05
10.	Subchronic	28	6	10.7	1–4	2.0±0.56		

Recovery group**								

a.	Control*	–	6	–	1–3	2.0±0.43	0	P>0.05
b.	Treated	–	6	10.7	2–2	2.0±0.0		

* Controls were given groundnut oil only

** Recovery group were given oil (a) and cypermethrin (b) treatment for 28 days and effects assessed after 60 days

**Table 8:** Effect of Cypermethrin on Total Leukocyte Count (thousand/mm^3^) in *Rattus norvegicus.*

S.No.	Treatment	Treatment duration (Days)	No. of rats treated	Dose (mg/kg b.w.)	Range	Mean±S.E.	% change	Significance level
1.	Control*	1	6	–	6.2–7.6	6.87±0.22	±44	P>0.05
2.	Acute	1	6	300	6.6–7.2	6.9±0.11		
3.	Control*	7	6	–	6.0–7.2	6.7±0.20	±4.48	P>0.05
4.	Subchronic	7	6	10.7	6.8–7.4	7.0±0.13		
5.	Control*	14	6	–	6.2–7.5	6.87±0.91	±17.61	P<0.001
6.	Subchronic	14	6	10.7	7.6–8.6	8.08±0.13		
7.	Control*	21	6	–	6.0–7.4	6.75±0.22	±13.77	P<0.01
8.	Subchronic	21	6	10.7	7.6–7.8	7.68±0.04		
9.	Control*	28	6	–	6.3–7.6	7.05±0.07	±12.76	P<0.01
10.	Subchronic	28	6	10.7	7.8–8.2	7.95±0.07		

Recovery group**								

a.	Control*	–	6	–	6.0–7.4	6.75±0.22	±1.78	P>0.05
b.	Treated	–	6	10.7	6.2–7.4	6.87±0.19		

* Controls were given groundnut oil only

** Recovery group were given oil (a) and cypermethrin (b) treatment for 28 days and effects assessed after 60 days

**Table 9:** Effect of Cypermethrin on Bone Marrow Counts for Myeloid-Erythroid ratio in *Rattus norvegicus.*

S.No.	Treatment	Treatment duration (Days)	No. of rats treated	Dose (mg/kg b.w.)	Myeloid			Erythroid			M-E Ratio
					Range	Mean±S.E.	Significance level	Range	Mean±S.E.	Significance level	
1.	Control*	1	6	–	70–77	74±0.23	P>0.05	20–32	26±0.76	P>0.05	2.84:1
2.	Acute	1	6	300	68–75	72±0.61		21–32	28±0.57		2.57:1
3.	Control*	7	6	–	64–80	75±0.77	P<0.05	22–29	25±0.98	P<0.05	3:1
4.	Subchronic	7	6	10.7	61–73	67±0.85		25–36	33±0.73		2.03:
5.	Control*	14	6	–	64–80	74±0.26	P<0.05	20–31	26±0.31	P<0.05	2.84:1
6.	Subchronic	14	6	10.7	58–69	65±0.53		31–39	35±0.24		1.85:1
7.	Control*	21	6	–	65–82	73±0.42	P<0.05	22–29	27±1.21	P<0.05	2.70:1
8.	Subchronic	21	6	10.7	54–65	61±0.96		25–42	39±0.64		1.56:1
9.	Control*	28	6	–	70–79	76±1.04	P<0.01	19–28	24±1.02	P<0.01	3.16:1
10.	Subchronic	28	6	10.7	48–57	55±0.75		38–49	45±0.43		1.2:1

Recovery group**											

a.	Control*	–	6	–	72–78	75±0.98	P>0.05	21–28	25±0.57	P>0.05	3:1
b.	Treated	–	6	10.7	71–80	76±1.10		20–26	24±0.98		3.16:1

* Controls were given groundnut oil only

** Recovery group were given oil (a) and cypermethrin (b) treatment for 28 days and effects assessed after 60 days

**Table 10:** Effect of Cypermethrin on Differential Leukocyte Count in *Rattus norvegicus.*

Cell type	Treatment	No. of days	Control*		Treatment		% Change	Significance
			Range	Mean±S.E.	Range	Mean±S.E.		
Neutrophils	Acute	1	24-28	26.0±0.78	23-27	25.28±1.23	-0.46	P>0.05
	Subchronic	7	26-30	28.22±1.73	28-41	30.12±2.60	±6.73	P<0.05
		14	23-29	26.00±1.73	32-41	36.66±2.60	±41	P<0.05
		21	28-30	29.33±0.67	33-36	34.33±1.2	±17.05	P<0.01
		28	27-30	28.22±0.63	29-33	32.0±1.42	±13.39	P<0.01
	Recovery**	60	25-28	27.10±0.75	24-27	26.87±0.65	-0.52	P>0.05
Eosinophils	Acute	1	2-4	3.30±0.75	2-5	4.04±0.64	±32.89	P>0.05
	Subchronic	7	2-3	2.21±0.47	1-3	2.46±0.07	±11.31	P>0.05
		14	1-3	1.93±0.86	2-4	2.34±0.98	±21.24	P>0.05
		21	1-3	2.00±0.54	1-3	2.00±0.54	-	P>0.05
		28	2-3	2.65±1.16	1-3	2.00±0.65	-24	P>0.05
	Recovery**	60	2-4	2.28±0.64	1-3	1.69±0.81	-28.99	P>0.05
Lymphocytes	Acute	1	65-73	70.08±0.57	67-72	70.68±1.31	-0.28	P>0.05
	Subchronic	7	67-71	69.57±0.45	66-70	67.42±0.72	-3.09	P>0.05
		14	67-72	71.40±1.64	58-63	61.0±0.89	-14.56	P>0.05
		21	67-70	68.67±0.88	63-64	63.67±0.58	-7.06	P<0.01
		28	66-70	69.13±0.65	63-68	64.25±1.63	-7.06	P<0.01
	Recovery**	60	65-72	70.62±0.97	66-72	70.70±1.20	±0.13	P>0.05
Monocytes	Acute	1	00-01	0.68±0.35	0	0	0	No change
		7	00	0	0	0	0	No change
	Subchronic	14	0-1	0.62±0.33	1-2	1.03±0.74	±0.53.73	P>0.05
		21	0	0	0	0	0	No change
		28	0	0	0	0	0	No change
	Recovery**	60	0	0	0	0.73±0.03	-	P>0.05

* Controls were given groundnut oil only

** Recovery group were given oil (a) and cypermethrin (b) treatment for 28 days and effects assessed after 60 days

### Qualitative changes

Qualitative changes in blood included crenated, hypochromatic red blood cells, microcystosis, appearance of stomatocytes, poikilocytosis, giant platelet formation and hyepersegmented neutrophils (Plate I–X), whereas those of bone marrow included increased appearance of immature haematological precursors such as orthochromatic and polychromatic erythroblasts, megaloblasts, myeloblasts, polymorphonuclear neutrophils (Plate XI–XVII).

**Plate I:**
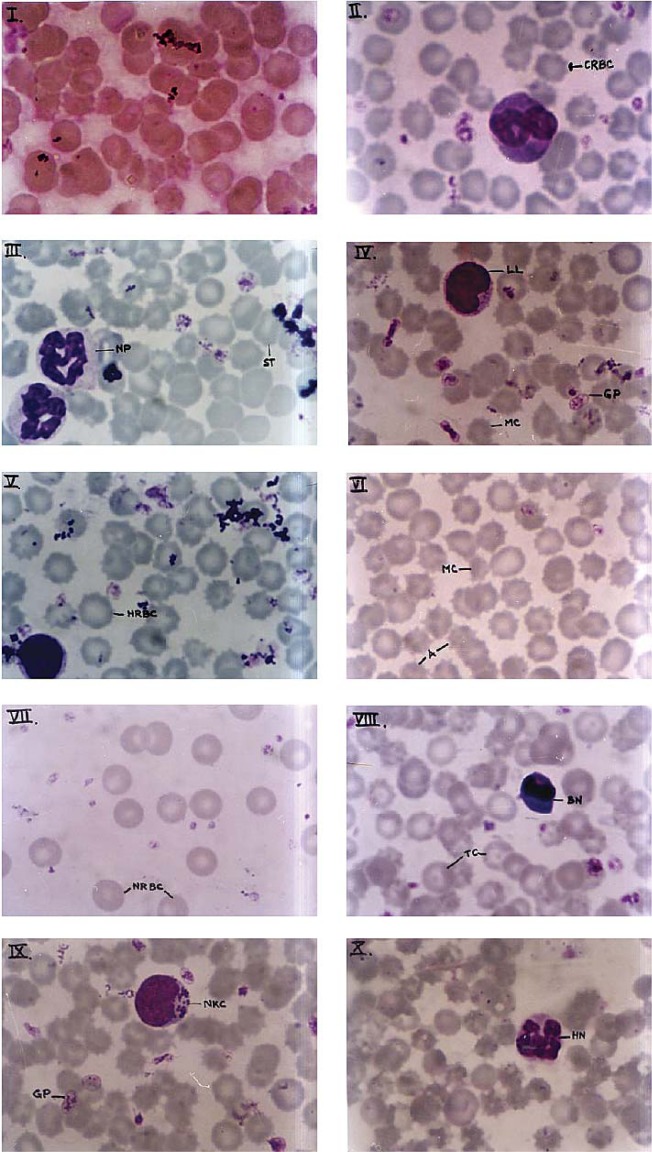
Photomicrograph of peripheral blood film of *Rattus norvegicus* of control group showing normal red blood cells.

**Plate II:**
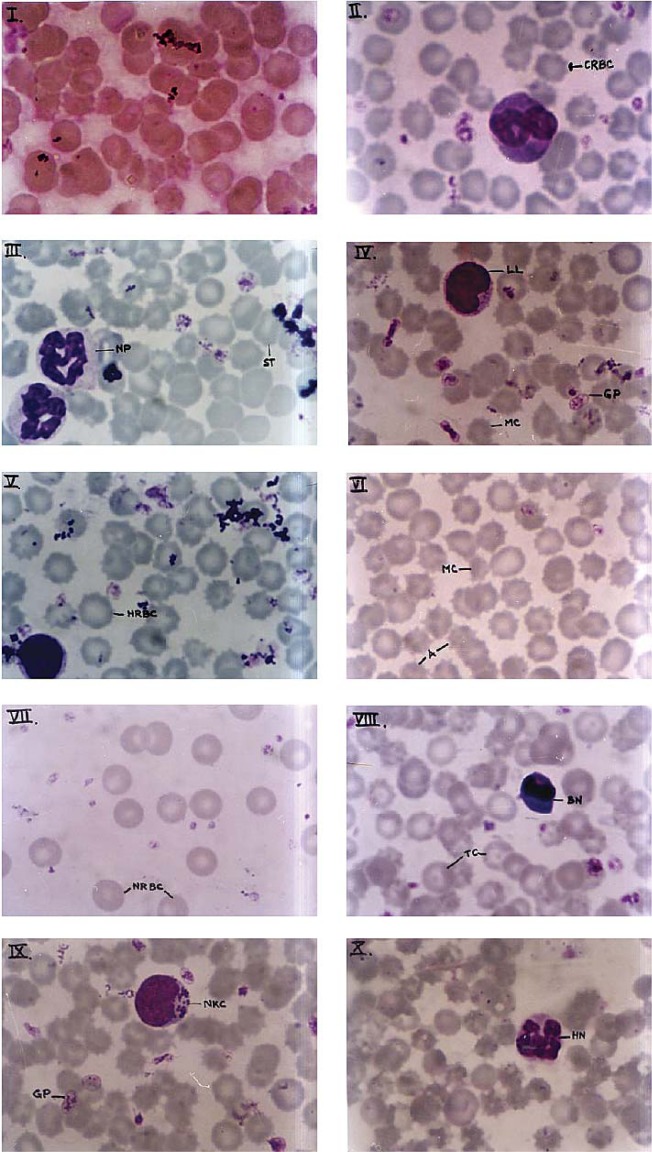
Photomicrograph of peripheral blood film of *Rattus norvegicus* after acute (1 day) cypermethrin treatment showing crenated red blood cells (CRBC).

**Plate III:**
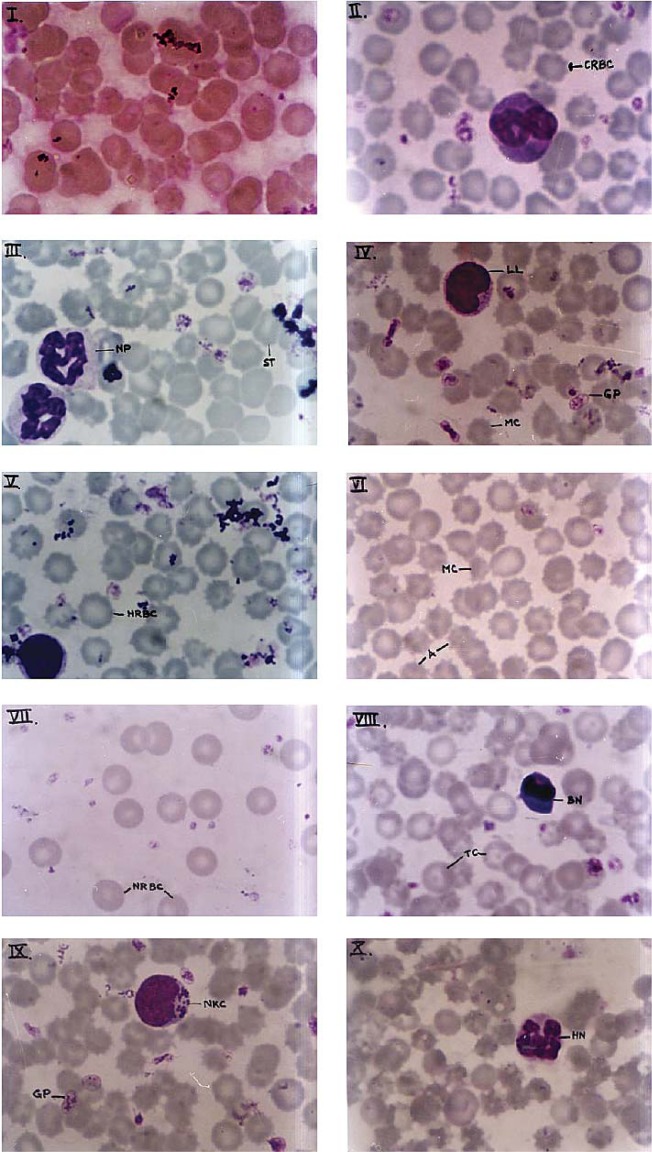
Photomicrograph of peripheral blood film of *Rattus norvegicus* after 7 days of subchronic treatment with cypermethrin showing stomatocyte (ST) and normal neutrophils (NP).

**Plate IV:**
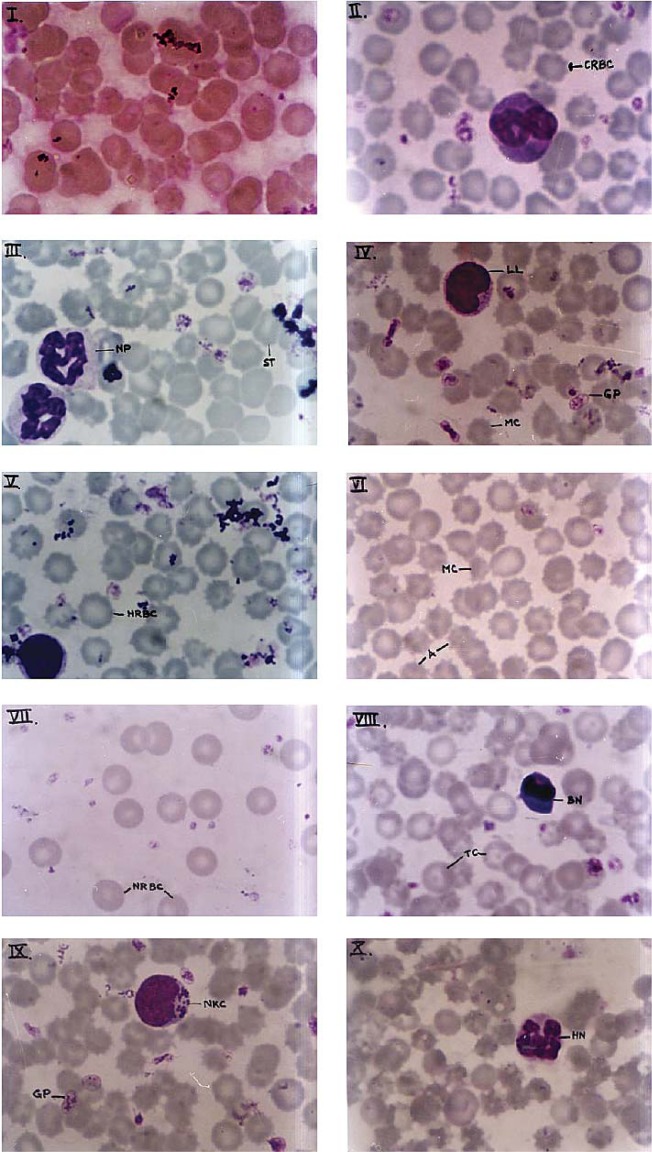
Photomicrograph of peripheral blood film of *Rattus norvegicus* after 14 days of subchronic treatment with cypermethrin showing giant platelet (GP), large lymphocyte (LL) and microcytes (MC).

**Plate V:**
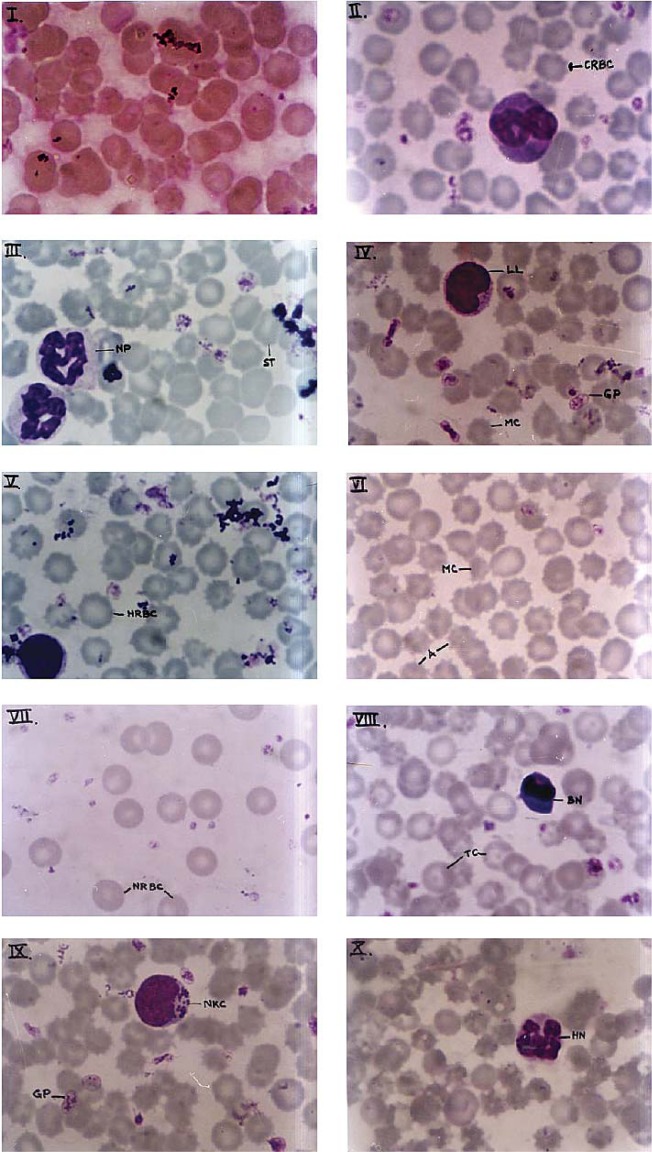
Photomicrograph of peripheral blood film of *Rattus norvegicus* after 21 days of subchronic treatment showing hypochromatic red blood cell (HRBC).

**Plate VI:**
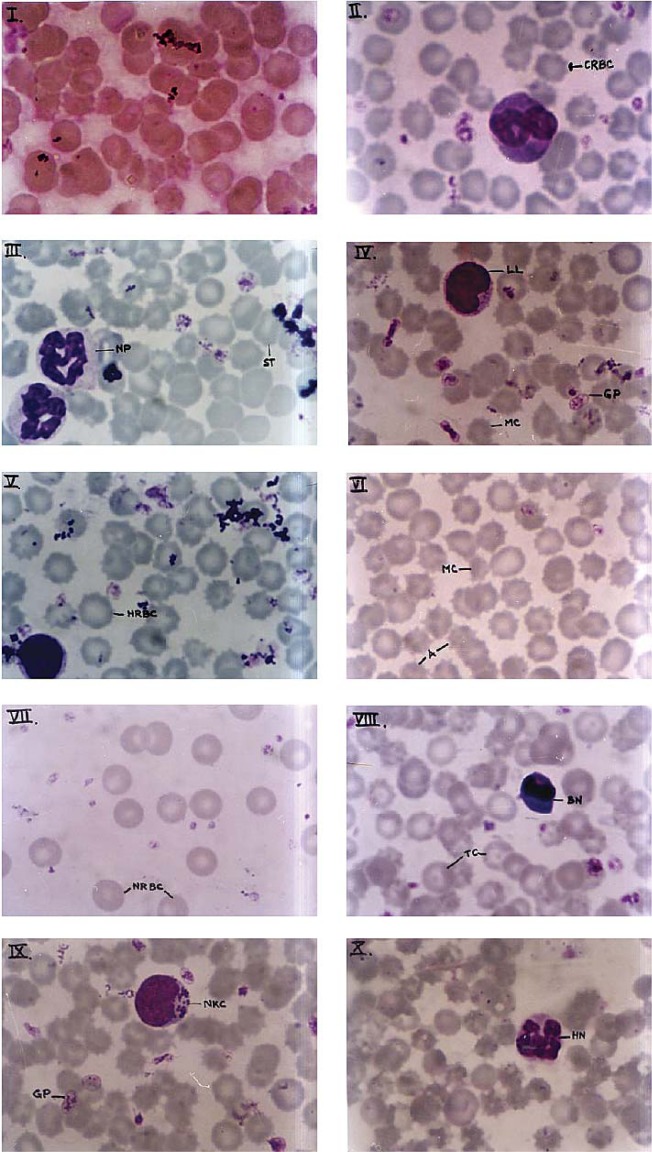
Photomicrograph of peripheral blood film of *Rattus norvegicus* after 28 days of subchronic treatment with cypermethrin showing microcytes (MC) and acanthocytes (A).

**Plate VII:**
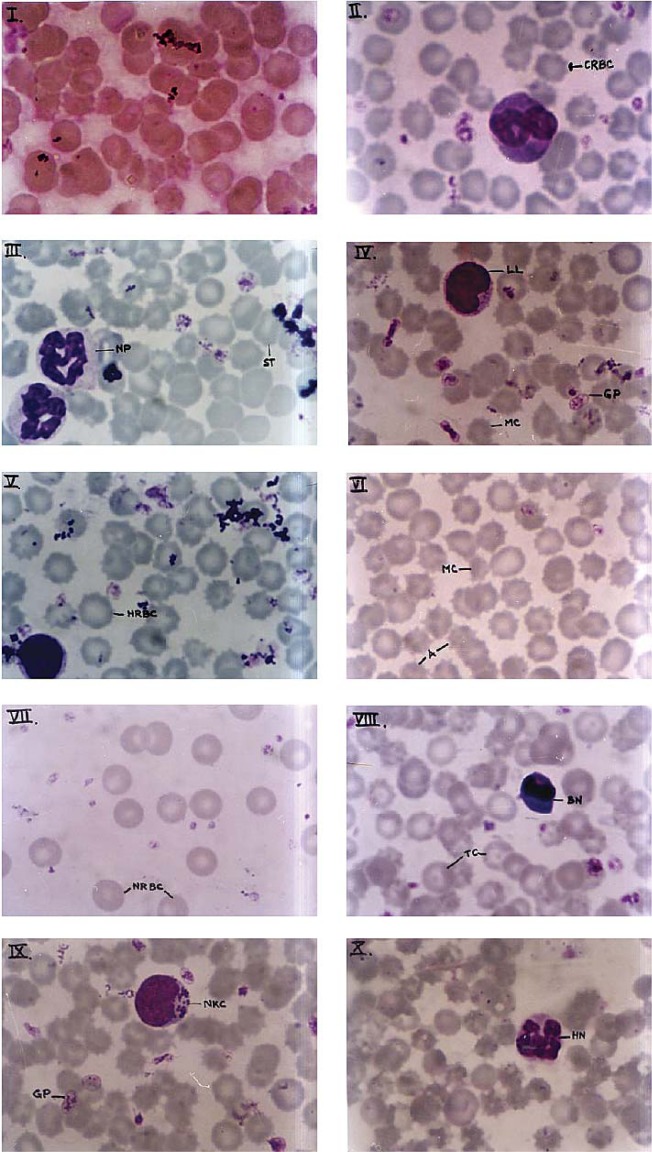
Photomicrograph of peripheral blood film of *Rattus norvegicus* of recovery group showing appearance of normal red blood cells (NRBC).

**Plate VIII:**
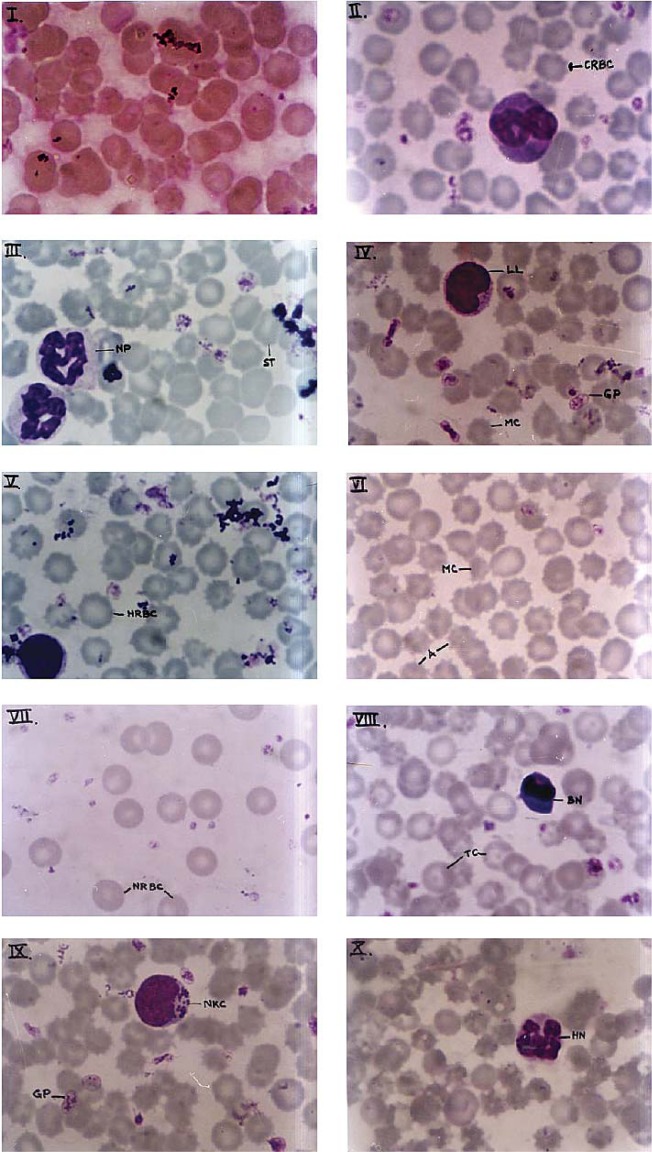
Photomicrograph of peripheral blood film of *Rattus norvegicus* after cypermethrin treatment showing target cell (TC) and basophilic normoblast (BN).

**Plate IX:**
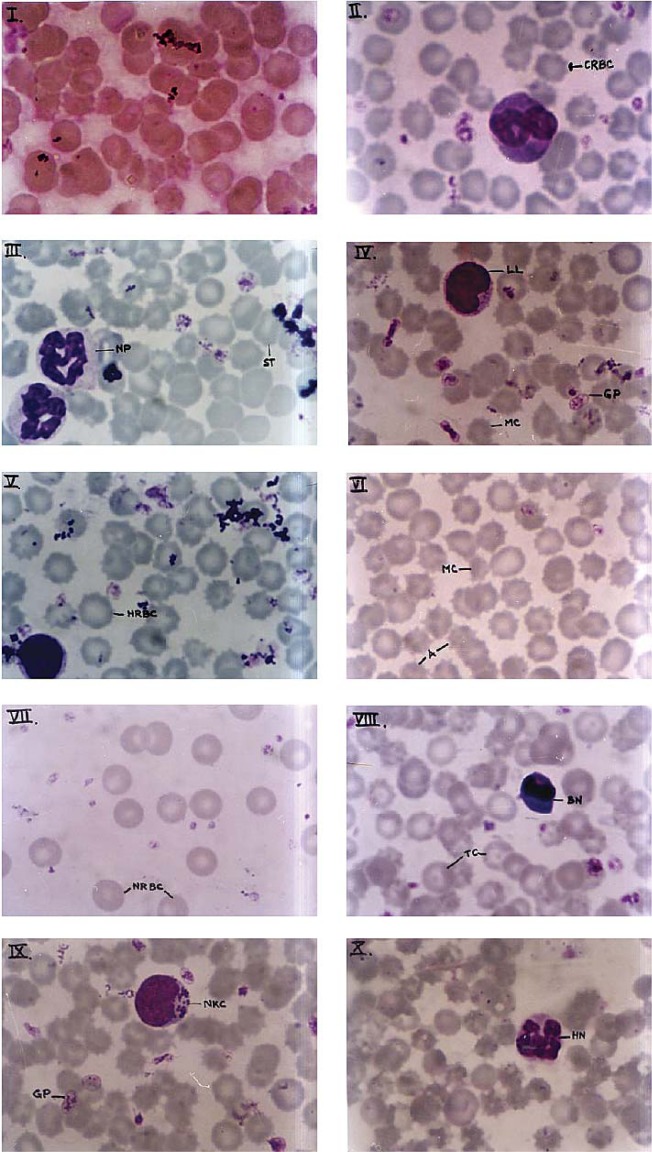
Photomicrograph of peripheral blood film of *Rattus norvegicus* after cypermethrin treatment showing natural killer cell (NKC) and giant platelet (GP).

**Plate X:**
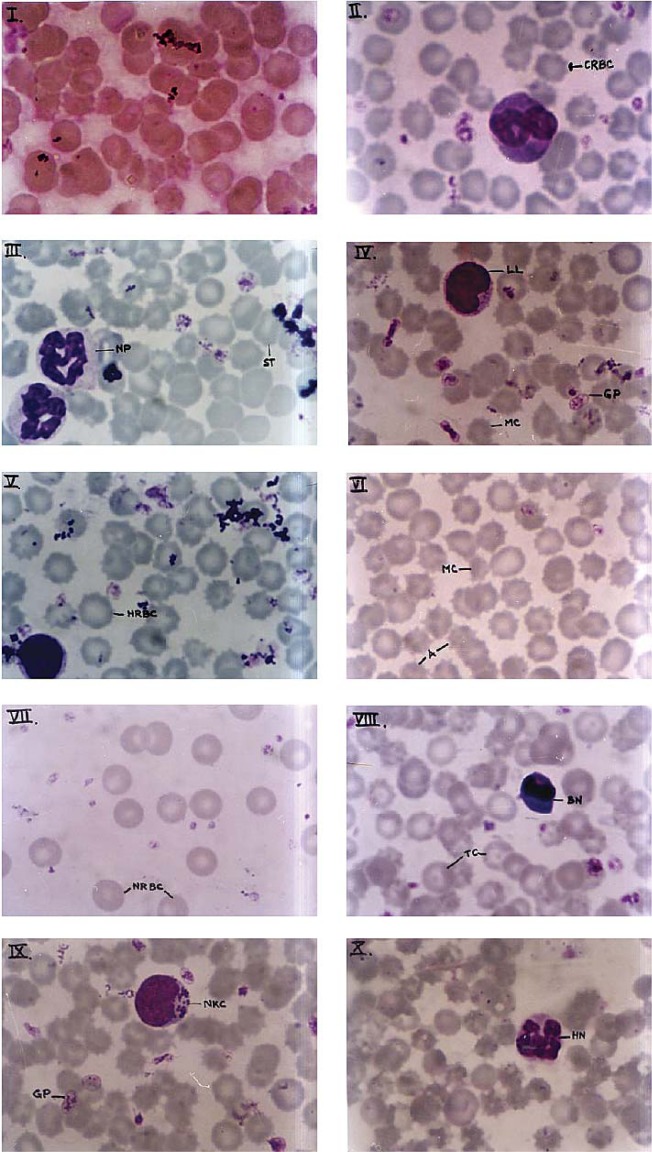
Photomicrograph of peripheral blood film of *Rattus norvegicus* after cypermethrin treatment showing hypersegmented neutrophil (HN).

**Plate XI:**
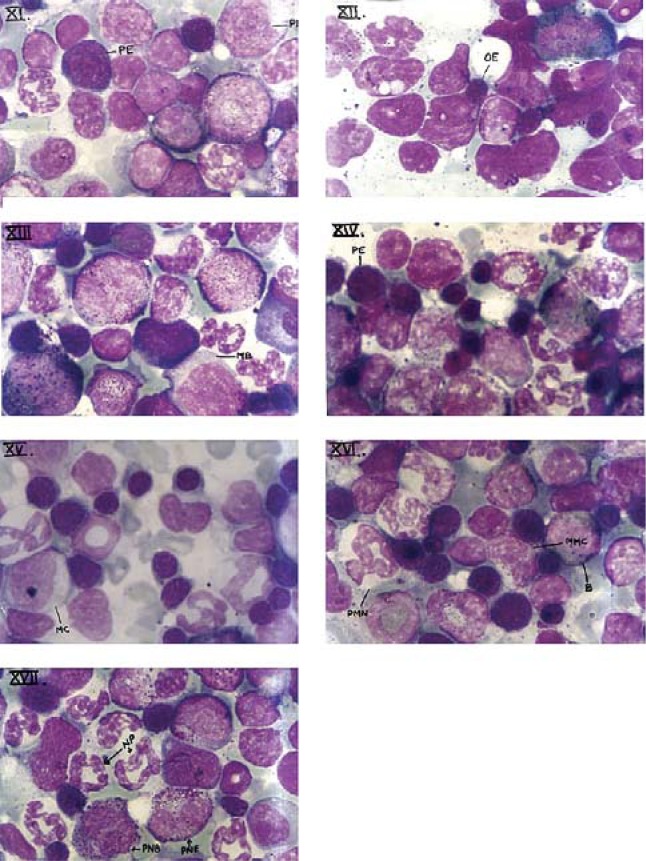
Photomicrograph of bone marrow smear of *Rattus norvegicus* of control group depicting promyelocyte (PMC) and polychromatic erythroblast (PE).

**Plate XII:**
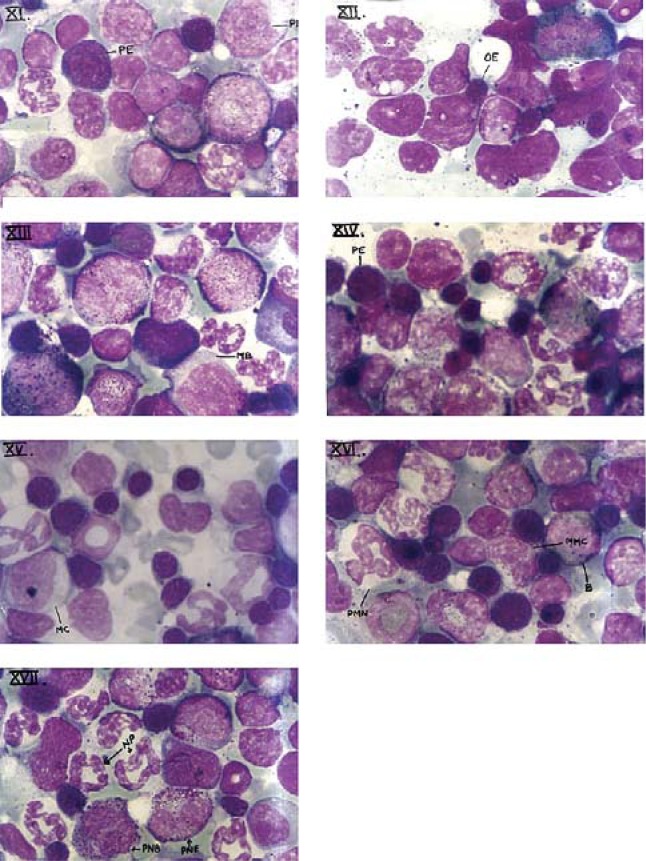
Photomicrograph of bone marrow smear of *Rattus norvegicus* after acute cypermethrin treatment showing orthochromatic erythroblast (OE).

**Plate XIII:**
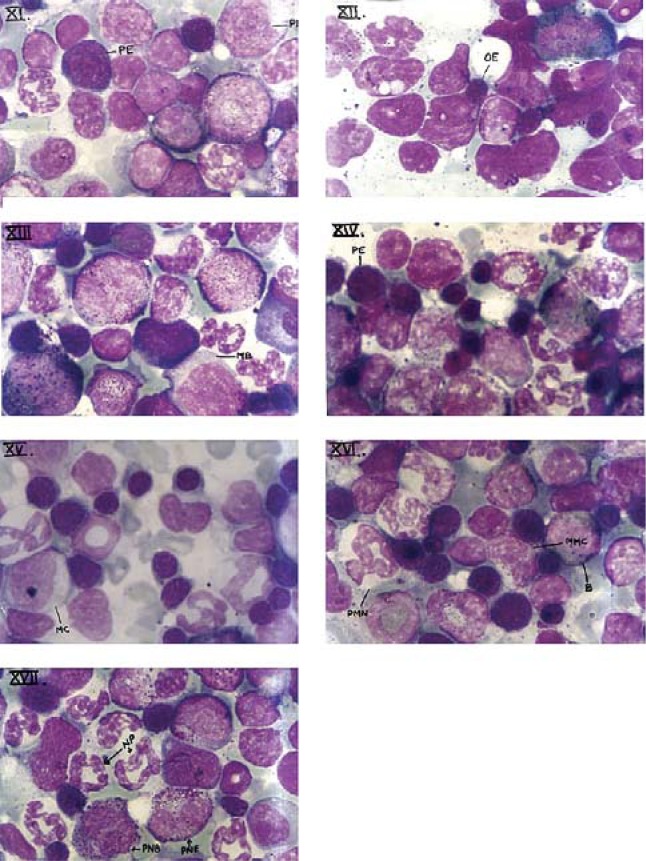
Photomicrograph of bone marrow smear of *Rattus norvegicus* after 7 days of cypermethrin treatment showing megaloblast (MB) and slight increase in cells of erythroid series.

**Plate XIV:**
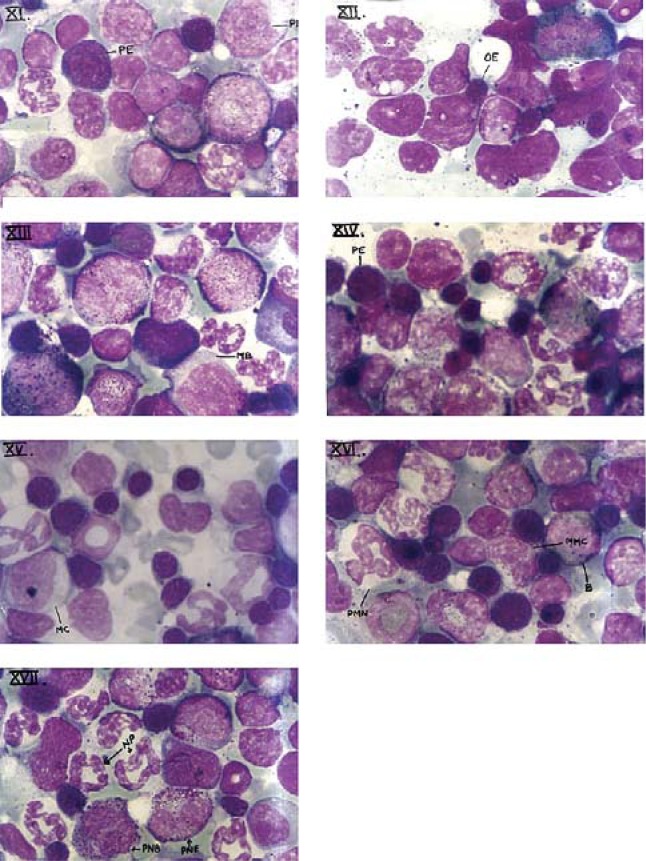
Photomicrograph of bone marrow smear of *Rattus norvegicus* after subchronic (14 days) cypermethrin treatment showing polychromatic erythroblast (PE) and increase in erythroid precursors.

**Plate XV:**
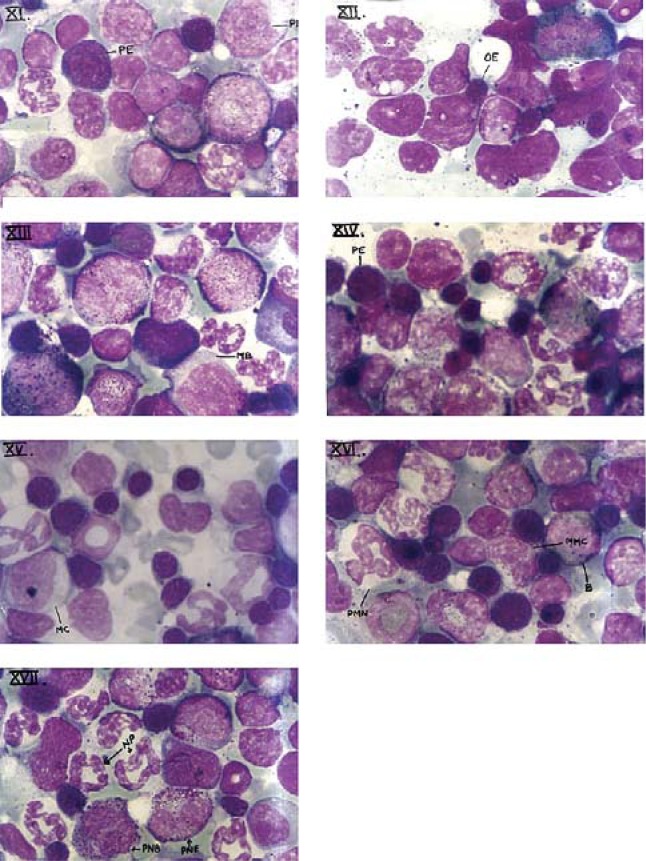
Photomicrograph of bone marrow smear of *Rattus norvegicus* after subchronic (21 days) cypermethrin treatment depicting myelocyte (MC) and moderate increase in erythroid precursors.

**Plate XVI:**
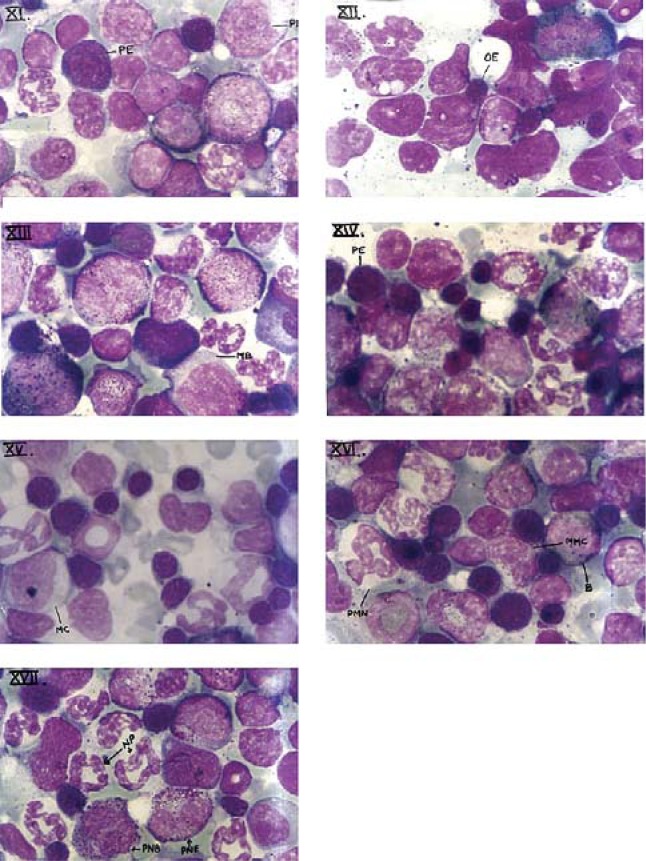
Photomicrograph of bone marrow smear of *Rattus norvegicus* after subchronic (28 days) treatment showing metamyelocyte (MMC) band stage (B), polymorphonuclear neutrophils (PMN) and highly increased proportion of erythroid cells.

**Plate XVII:**
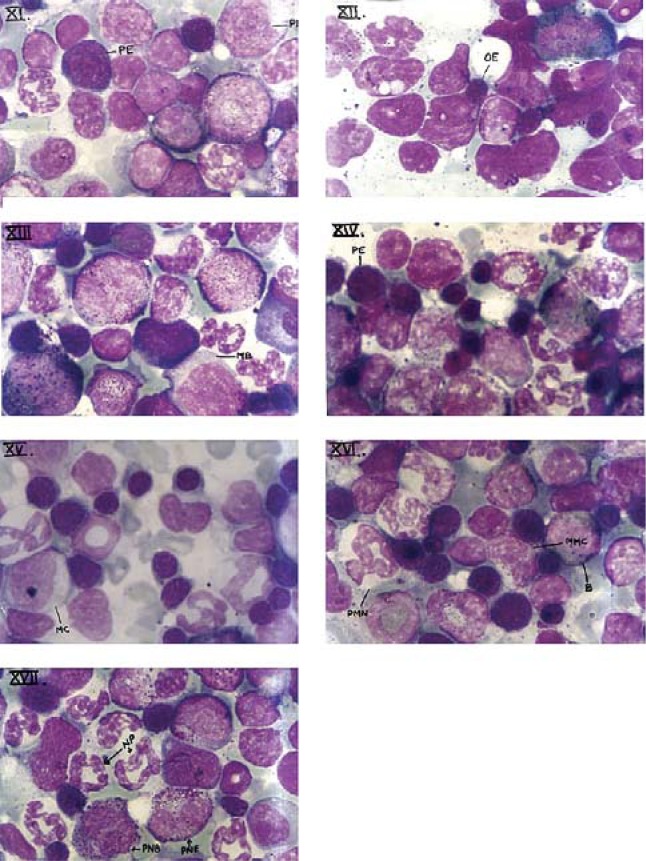
Photomicrograph of bone marrow smear of *Rattus norvegicus* of recovery group (60 days) showing developing neutrophils (NP), polymorphonuclear eosinophil (PNE) and polymorphonuclear basophil (PNB).

## Discussion

Haematological evaluation is one of the best methods to assess toxicity of pyrethroids. Pyrethroids can induce toxicity to various haematological components in two ways, either by interference with mature components flowing in peripheral blood or with development of such integral blood components (Pande, 2001; Saka *et al.,* 2011; Bhushan *et al.,* 2013b).

Of the circulating elements of blood, a decrease found in total red cell count in the present study following cypermethrin intoxication is an indicator of interference of the experimental compound as well as its metabolically broken-down byproducts. This interference may be in the form of destruction of the normal erythrocytic membrane structure causing lysis of erythrocytes resulting in this decrease (Sakoori *et al.,* 1990; Ahmad *et al.,* 2009; Abbassy & Mossa, 2012). Pyrethroids, including cypermethrin, are also capable of causing alterations in blood forming organs of albino rats (Singh & Saxena, 2002; Assayed *et al.,* 2010; Lamfon, 2013). Qualitatively, stomatocytes, hypochromasia and poikilocytosis seen in blood smears of cypermethrin intoxicated rats along with the presence of various immature erythrocytic precursors in bone marrow smears, are indicative of haemolysis and erythrocytic membrane abnormalities as well as of bone marrow dysfunction. Reduction in the number of RBCs is thus an outcome of haemolysis of red blood cells observed qualitatively and quantitatively as well as of a possible dysfunction of bone marrow, which was apparent qualitatively.

Further, a significant decrease in RBC count was also accompanied with decrease in haemoglobin concentration following cypermethrin intoxication in the present investigation. Microcytosis and hypochromasia, apparent qualitatively, is an indicator of impaired haemoglobin synthesis. Therefore decrease in haemoglobin concentration registered under pyrethroid stress is the result of a reduction in the number of red blood cells due to erythrocytopenia and impaired haemoglobin synthesis in the erythroblasts (Sakoori *et al.,* 1990; Jain *et al.,* 2009; Abbassy & Mossa, 2012).

Decrease in PCV, as reported in the present study, can also be correlated with reduced RBC count coupled with hypohaemoglobinaemia accompanied by hypoplasia of bone marrow and dilutional anaemia (Pande, 2001; Jain *et al.,* 2009; Abbassy & Mossa, 2012).

The mean corpuscular volume (MCV), mean corpuscular haemoglobin (MCH) and mean corpuscular haemogloin concentration (MCHC) are generally referred to as absolute values. These values indicate abnormalities in the erythrocytes and their calculation is widely used in the classification of anaemia under pathological conditions (Dacie & Lewis, 1991). A significant decline in MCV is also an outcome of cypermethrin induced toxicity to bone marrow which is producing microcytes along with exosmosis due to electrolyte imbalance. Decrease in the value of MCH may be attributed to microcytic anaemia following cypermethrin intoxication quantitatively and hypochromasia qualitatively.

Non-significant but generalised decrease in MCHC value seems to be the result of microcytosis of red blood cells. Since MCHC represents inverse relationship with PCV and haemoglobin levels, a decrease in values of both these parameters in intoxicated experimental rats nullified the effect of eachothers and thus MCHC showed a non-significant effect (Pande, 2001).

ESR values were also elevated non-significantly, which however is an indicator of inflammatory manifestation and gains support by rise in TLC following cypermethrin intoxication. Leukocytosis may be regarded as the body's response to the invasion of a foreign substance into the body (Saxena & Tomar, 2003). Further, increase in TLC in the present study was mainly due to the increase in neutrophils, which are granulocytes of bone marrow origin. Increased neutrophil count may be an outcome of stimulated granulocyte precursors to produce more and more of these cells, while cytotoxic effects of cypermethrin on the spleen, the site where lymphocytes are formed, resulted in lymphoma in cypermethrin treated rats. The rise in TLC may be due to qualitative responses such as hyper-segmentation in neutrophils, a consequence of disordered granulopoiesis, presence of natural killer cells in peripheral blood and formation of giant platelets.

Qualitatively, microcytosis, poikilocytosis, stomatocytosis, hypochromasia, hypersegmentation and giant platelet formation in peripheral blood and the presence of various altered precursors in bone marrow smears are strong indicators of the haematotoxic potential of cypermethrin at both functional and developmental levels of blood components.

Further the reversal of altered values towards normalcy after the recovery phase of twenty-two days points to a time-based process.

## References

[CIT0001] Abbassy MA, Mossa ATH (2012). Haemato-biochemical effects of formulated and technical cypermethrin and deltamethrin insecticides in male rats. J Pharmacol Toxicol.

[CIT0002] Addy-Orduna LM, Maria-Elena Z, Canavelli SB, Mineau P (2011). Formulated beta-cyfluthrin shows wide divergence in toxicity among bird species. J Toxicol.

[CIT0003] Ahmad L, Khan A, Khan MZ, Hussain I (2009). Cypermethrin induced anaemia in male rabbits. Pakistan Vet J.

[CIT0004] Aldana L, Tsutsumi V, Craigmill A, Silveira MI, de Mejia EG (2001). Alpha-tocopherol modulates liver toxicity of the pyrethroid cypermethrin. Toxicol Lett.

[CIT0005] Assayed ME, Khalaf AA, Salem HA (2010). Protective effects of garlic extract and vitamin C against in vivo cypermethrin-induced cytogenetic damage in rat bone-marrow. Mut Res.

[CIT0006] Bhushan B, Saxena N, Saxena PN (2010). Beta-cyfluthrin induced histochemical alterations in the liver of albino rat. Scand J Lab Anim Sci.

[CIT0007] Bhushan B, Saxena PN, Saxena N (2013a). Biochemical and histological changes in rat liver caused by cypermethrin and beta-cyfluthrin. Arch Indus Hyg Toxicol.

[CIT0008] Bhushan B, Pande S, Saxena N, Saxena PN (2013b). Serum biochemical responses under stress of cypermethrin in albino rat. Env Exp Biol.

[CIT0009] Bhushan B, Prabhu NS, Saxena N (2013c). Histochemical changes in rat liver under stress of type II pyrethroids. Int J Adv Res Tech.

[CIT0010] Corcellas C, Feo ML, Torres JP, Malm O, Ocamp-Deque W, Eljarrat E, Barcelo D (2012). Pyrethroids in human breast milk. Occurrence and nourishing daily intake estimation. Env Int.

[CIT0011] Dacie JV, Lewis MS (1991). Practical haematology.

[CIT0012] Finey DJ (1971).

[CIT0013] Jain N, Sharma P, Sharma N, Joshi SC (2009). Haemato-biochemical profile following subacute toxicity of malathion in male albino rats. Pharmacologyonline.

[CIT0014] Lamfon HA (2013). Modulatory effect of vitamin E against fenvalerate induced immunotoxicity in albino mice. Int J Immuno Res.

[CIT0015] Pande S (2001). Effect of synthetic pyrethroids on certain haematobiochemical parameters of *Rattus norvegicus*.

[CIT0016] Rana N, Saxena N, Sharma HN, Saxena PN (2008). Comparative genotoxicity of alpha-cyano pyrethroids on *Drosophila melanogaster*. Entamon.

[CIT0017] Saka WA, Akhigbe RE, Azeez OM, Babatunde TR (2011). Effect of pyrethroid insecticide exposure on haematological and haemostatic profiles in rats. Pak J Biol Sci.

[CIT0018] Saxena PN, Tomar V (2003). Assessment of comparative heamatoxicity of Cybil and fenvalerate in *Rattus norvegicus*. Bull Environ Contam Toxicol.

[CIT0019] Sayim F, Yavasoglu NUK, Uyanikgil Y, Aktug H, Yavasoglu A, Turgut M (2005). Neurotoxic effects of cypermethrin in Wistar rats: a haematological, biochemical and histopathological study. J Hlth Sci.

[CIT0020] Shakoori AR, Aziz F, Alam J, Ali SS (1990). Toxic effects of talastar, a new synthetic pyrethroid, on blood and liver of rabbit. Pakistan J Zool.

[CIT0021] Singh VK, Saxena PN (2002). Genotoxic potential of cypermethrin in mammalian haemopoietic system. Him J Env Zool.

[CIT0022] Singh AK, Saxena PN, Sharma HN (2009). Stress induced by beta-cyfluthrin, a type-2 pyrethroid, on brain biochemistry of Albino rat (*Rattus norvegicus*). Biol Med.

[CIT0023] Soderlund DM, Clark JM, Sheets LP, Mullin LS, Piccirillo VJ, Sargent D, Stevens JT, Weiner ML (2002). Mechanisms of pyrethroid neurotoxicity: implications for cumulative risk assessment. Toxicology.

[CIT0024] Spencer CI, Sham JSK (2005). Mechanisms underlying the effects of the pyrethroid tefluthrin on action potential duration in isolated rat ventricular myocytes. J Pharmacol Exp Therap.

